# Application of multimodal data fusion and explainable AI for classifying water stress in sweet potatoes

**DOI:** 10.3389/fpls.2025.1681915

**Published:** 2025-10-07

**Authors:** Ji Won Choi, Soo Been Cho, Mohamad Soleh Hidayat, Woon-Ha Hwang, Young-Son Cho, Hoonsoo Lee, Byoung-Kwan Cho, Geonwoo Kim

**Affiliations:** ^1^ Department of Biosystems Engineering, College of Agricultural and Life Sciences, Gyeongsang National University, Jinju-si, Gyeongsangnam-do, Republic of Korea; ^2^ Division of Crop Production and Physiology, National Institute of Crop Science, Rural Development Administration, Wanju-gun, Republic of Korea; ^3^ Department of Smart Agro Industry, College of Life Science, Gyeongsang National University, Jinju, Republic of Korea; ^4^ Department of Biosystems Engineering, College of Agriculture, Life and Environment Sciences, Chungbuk National University, Cheongju-si, Chungbuk-do, Republic of Korea; ^5^ Department of Biosystem Machinery Engineering, Chungnam National University, Daejeon, Republic of Korea; ^6^ Institute of Agriculture and Life Sciences, Gyeongsang National University, Jinju-si, Gyeongsangnam-do, Republic of Korea

**Keywords:** thermal imagery (TRI), red-green-blue (RGB) imagery, sweet potato, water stress, artificial intelligence (AI)

## Abstract

Sweet potato (Ipomoea batatas L.) exhibits strong resilience in nutrient-poor soils and contains high levels of dietary fiber and antioxidant compounds. It also is highly tolerant to water stress, which has also contributed to its global distribution, particularly in regions prone to climatic variability. However, frequent abnormal climatic events have recently caused declines in both the quality and yield of sweet potatoes. To address this, machine learning (ML) and deep learning (DL) models based on a Vision Transformer–Convolutional Neural Network (ViT-CNN) were developed to classify water stress levels in sweet potato. RGB–thermal imagery captured from low-altitude platforms and various growth indicators were used to develop the classifier. The K-Nearest Neighbors (KNN) model outperformed other ML models in classifying water stress levels at all growth stages. The DL model simplified the original five-level water stress classification into three levels. This enhanced its sensitivity to extreme stress conditions, improve model performance, and increased its applicability to practical agricultural management strategies. To enhance practical applicability under open-field conditions, several environmental variables were newly defined to calculate the crop water stress index (CWSI). Furthermore, an integrated system was developed using gradient-weighted class activation mapping (Grad-CAM), explainable artificial intelligence (XAI), and a graphical user interface (GUI) to support intuitive interpretation and actionable decision-making. The system will be expanded into an online and fixed-camera platform to enhance its applicability to smart farming in diverse field crops.

## 1 Introduction

Sweet potato (*Ipomoea batatas L.*) is recognized as a nutritionally rich staple and is cultivated as an important food security crop in many countries due to its ability to grow well in poor soil conditions (Laveriano-Santos et al., 2022). Sweet potato is resilient to temporary drought and low-fertility soils, supporting consistent growth in poor climate conditions (Lindqvist-Kreuze et al., 2024; Yan et al., 2022). Owing to its adaptive capacity and nutritional value, sweet potato is globally regarded as a crop that can contribute to addressing food shortages and food security (Sapakhova et al., 2023).

The yield and quality of sweet potato, however, have been considerably affected by frequent abnormal climate events (Pushpalatha and Gangadharan, 2024). This has led to substantial economic losses for many farming households (Tedesco et al., 2023). Prolonged drought or waterlogging can inhibit the normal growth of sweet potato. In particular, insufficient water supply during the tuber formation stage can lead to inadequate accumulation of starch and fructose within the tubers, resulting in reduced yield and deteriorated quality (Zhang et al., 2002; Nedunchezhiyan et al., 2012). Water saturation during critical growth stages, particularly tuber initiation, interferes with carbohydrate translocation and accumulation, which leads to suboptimal yield and compromised quality (Gouveia et al., 2020).

Crop water stress traditionally has been evaluated through direct measurement of photosynthetic activity under field conditions or by monitoring soil moisture levels in conjunction with meteorological parameters (Ihuoma and Madramootoo, 2017). However, such methods are often labor-intensive and time-consuming and consequently are not practical for large-scale or continuous monitoring (Yang et al., 2020). Furthermore, these approaches are frequently constrained by subjectivity in data collection and interpretation, which may lead to inconsistencies between observers and experimental conditions (Grassini et al., 2015). In light of this, there is a growing need to develop new technologies that can overcome the limitations of traditional methods and enable more efficient and accurate assessment of water stress (Kamarudin et al., 2021). Recently, the integration of remote sensing technologies, sensor-based measurements, and artificial intelligence (AI) analysis techniques in agricultural field has made it possible to monitor crop growth conditions rapidly, precisely, and at early stages (Wei et al., 2023).

Among these approaches, unmanned aerial vehicle(UAV)-based multispectral and hyperspectral image analysis is commonly used to assess crop growth characteristics (Wang et al., 2018; Oré et al., 2020). The operation of precision agriculture systems often requires a substantial level of technical knowledge in hardware calibration, data analysis, and model interpretation. This dependency on skilled personnel limits the practical usability of such technologies for general farmers who may lack formal training or access to technical support and thus widens the digital divide in agriculture (ÇOBAN and OKTAY, 2018). In particular, the need for expensive equipment and complex data analysis procedures impede the adoption of such technologies by small- and medium-scale farms (Bhargava et al., 2024).

In addition, UAV-based high-altitude image acquisition is highly sensitive to external environmental factors, which imposes limitations on the precise measurement of subtle phenotypic traits in crops (Aburaed et al., 2023). It hence becomes challenging to obtain high-resolution data that capture subtle changes in leaves or reflect the characteristics of individual plants, and the spatial resolution of the collected data may be reduced in some environments (Lu et al., 2020).

Numerous studies have been conducted on the use of low-altitude imaging techniques to mitigate the limitations associated with conventional UAV-based approaches and to address the aforementioned constraints in quantitatively assessing crop water stress (Samseemoung et al., 2012; Sankaran et al., 2015). Low-altitude imaging enables the acquisition of more precise data due to its closer proximity to the crop canopy (Zhang et al., 2014). It is also well-suited for accurately analyzing subtle growth changes and the degree of water stress in individual plants (Huang et al., 2016). From a cost perspective, low-altitude imaging is advantageous compared to UAV-based approaches (Jin et al., 2017). Because it does not require specialized flight control skills and can be implemented using relatively inexpensive equipment, it is readily adoptable by farmers (Liu and Pattey, 2010).

In particular, the use of thermal imaging (TRI) cameras enables continuous and repeated collection of crop-level temperature data and thus is useful for acquiring time-series information on individual plants (Ishimwe et al., 2014). In addition, red-green-blue (RGB) cameras can be employed to evaluate plant status by capturing visual indicators such as color, brightness, and texture and thereby detect surface-level physiological changes (Vadivambal and Jayas, 2011; Messina and Modica, 2020).

The crop water stress index (CWSI) is becoming a representative indicator for quantitatively assessing the level of water stress experienced by crops (Idso et al., 1981). Calculated based on the difference between the actual observed canopy temperature and the theoretically possible maximum and minimum canopy leaf surface temperatures, the CWSI comprehensively reflects soil moisture conditions, weather variables, and the plant’s transpiration capacity (Trout et al., 2025; Katimbo et al., 2022). It can be effectively used in agricultural decision-making processes, such as determining optimal irrigation timing and optimizing water management, and also allows early detection of crop stress (Kullberg et al., 2024).

The accuracy of CWSI, however, relies on precise canopy temperature measurements, which require a range of specialized equipment and environmental conditions, including high-resolution thermal cameras, meteorological sensors, and calibration algorithms (Sánchez-Piñero et al., 2022). Due to these technical and economic constraints, practical application in typical cultivation fields or small-scale farms remains challenging (Wang et al., 2005). Accordingly, recent studies have explored the possibility of indirectly estimating canopy temperature or redefining the CWSI as a target variable for model training (Kamankesh and Ghayedi, 2023).

AI, particularly machine learning (ML) and deep learning (DL) techniques, has been actively utilized in agricultural decision-making (Ayoub Shaikh et al., 2022; Alibabaei et al., 2022). ML is effective for real-time analysis of crop growth conditions and yield prediction by integrating data from satellites, UAVs, RGB and TRI, internet of things (IoT) sensors, and meteorological information (Rahmani et al., 2021). This approach provides precise detection of water stress, temperature fluctuations, and abnormal growth patterns (Chlingaryan et al., 2018). DL can recognize complex patterns and handle large-scale data, and this allows it to capture nonlinear relationships and subtle environmental variables (Joshi et al., 2023). Through iterative learning, its accuracy improves over time, and it demonstrates high adaptability to various crops and environmental conditions (Meghraoui et al., 2024; Wang et al., 2022). As a result, it provides real-time decision-making, labor reduction, and improvements in productivity and quality, which in turn can lead to precise and automated agricultural practices (Tamayo-Vera et al., 2024).

This study developed ML classification models to assess the water stress levels of field-grown sweet potatoes based on leaf temperature and growth indicators obtained from low-altitude TRI and RGB imagery. In addition, DL classification models were constructed using TRI and RGB images. To enhance practicality under open-field cultivation conditions, the variables required for calculating the CWSI were replaced with field-observable variables, and a redefined formula for index computation was proposed. The ML models included logistic regression (LR), random forest (RF), k-nearest neighbors (KNN), multilayer perceptron (MLP), and support vector machine (SVM). For DL, a convolutional neural network (CNN) integrated with a vision transformer (ViT) was implemented. The performance of all models was evaluated and compared based on accuracy and K-fold cross-validation. A GUI-based system, termed the sweet potato water monitor system, was ultimately developed to classify water stress levels in sweet potato crops and to provide corresponding management recommendations, based on trained ML and DL models.

## 2 Materials and methods

### 2.1 Sample preparation

The cultivar used in this study was Jinyulmi and the experimental field, comprising two plots of 320 m² each (8 m × 40 m), was established at Gyeongsang National University’s Naedong campus. Sweet potato transplanting began in May 2024 using seedlings approximately 25–30 cm in length, and harvesting was conducted in September 2024. To help establish seedlings after transplanting, sufficient irrigation was provided for three weeks. Subsequently, RGB and thermal images of the sweet potato plants were acquired for analysis.

Soil moisture levels were categorized into five classes: Severe Dry (SD), Dry (D), Optimal (O), Wet (W), and Severe Wet (SW). These categories were defined based on volumetric water content (VWC) as follows: SD (≤10%), D (20 ± 2%), O (30 ± 3%), W (40 ± 3%), and SW (50%) (Huang et al., 2024). Approximately 300 samples were used in total, with around 50 samples per treatment group.

Irrigation was conducted using a subsurface drip irrigation system, and soil moisture content was measured at a depth of 20 cm using a portable soil moisture meter (FieldScout TDR-300, Spectrum Technologies, USA).

Sweet potato growth was assessed twice during each of the major growth stages: root differentiation, tuber initiation, bulking, and final harvest. To comprehensively evaluate crop growth conditions, stem length, normalized difference vegetation index (NDVI), chlorophyll fluorescence (CF), and SPAD values were collected as growth indicators. Stem length is a fundamental morphological indicator that indirectly reflects biomass accumulation and growth inhibition under water stress conditions (Fujii et al., 2014). NDVI, a representative remote sensing index based on photosynthetic activity, quantitatively indicates crop vigor and health and is highly sensitive to stress signals such as water deficiency or growth imbalance (Huang et al., 2021). CF is a physiological indicator capable of detecting functional abnormalities in the photosynthetic system and is useful for identifying early, non-visible stress responses. SPAD values indirectly estimate chlorophyll content in leaves, thereby reflecting photosynthetic capacity and nitrogen status (Gorbe and Calatayud, 2012; Uddling et al., 2007). By integrating these diverse growth indicators, the water stress responses of sweet potato were analyzed and used as input variables for the ML classifiers.

### 2.2 TRI and data acquisition

In the current study, Thermal images were acquired using an FLIR A65, a handheld infrared thermal camera equipped with a 640 × 512pixel microbolometer sensor. This device functions as a TRI temperature sensor capable of visually and comprehensively monitoring temperature variations. The acquired thermal images were calibrated using FLIR Tools, software provided by FLIR. To ensure the accuracy of temperature measurements of the leaves and plant canopy, the emissivity was set to 0.9 (Muller et al., 2021).

Thermal image acquisition was conducted manually from approximately 1.05 meters above the plant canopy height. To minimize the effects of direct sunlight, images were captured between 6:00 and 7:00 a.m. from the center of each crop plot. The collected images were then analyzed to determine the mean temperature and temperature distribution for each treatment group. Additionally, a calibration process was performed to ensure the accuracy of the infrared data captured through the camera lens. For this, the device’s performance was verified using ice (0 °C) and boiling water (100 °C), followed by the application of the automatic calibration function provided by the manufacturer through the FLIR Tools software (Swamidoss et al., 2021).

In this study, TRI was utilized in two ways. First, leaf temperature extracted from thermal images was used as a key indicator indirectly reflecting the plant’s physiological water status. Leaf temperature was integrated with other growth indicators, such as SPAD and the CWSI, as input variables for the ML-based classification models. Additionally, leaf temperature was used as leaf surface temperature data in the process of redefining the CWSI formula for field applicability. Second, data were directly utilized as input features for a CNN-based ViT classification model, in conjunction with RGB images. This approach was adopted to improve classification accuracy by integrating multimodal image information. The model is designed to enhance water stress classification performance and provide visual interpretability of water stress.

To correct for local temperature variations and measurement errors, leaf temperature was calculated based on the average of three manually selected, independent regions from each thermal image. These regions were morphologically distinct leaf areas, and the mean temperature of each was first computed. The final leaf temperature was then determined by averaging the values from the three regions. This strategy was employed to reduce noise caused by single-pixel extraction and to ensure that the resulting leaf temperature value reliably represented the overall leaf condition.

### 2.3 RGB data acquisition

In this study, an RGB image correction program was developed using Matlab R2024a (MathWorks, USA) to effectively compensate for color distortion and brightness irregularities that may occur during image acquisition. For color calibration, the international standard reference target color checker (X-Rite, USA) was employed to ensure color accuracy in the captured images.

**Figure 1:**
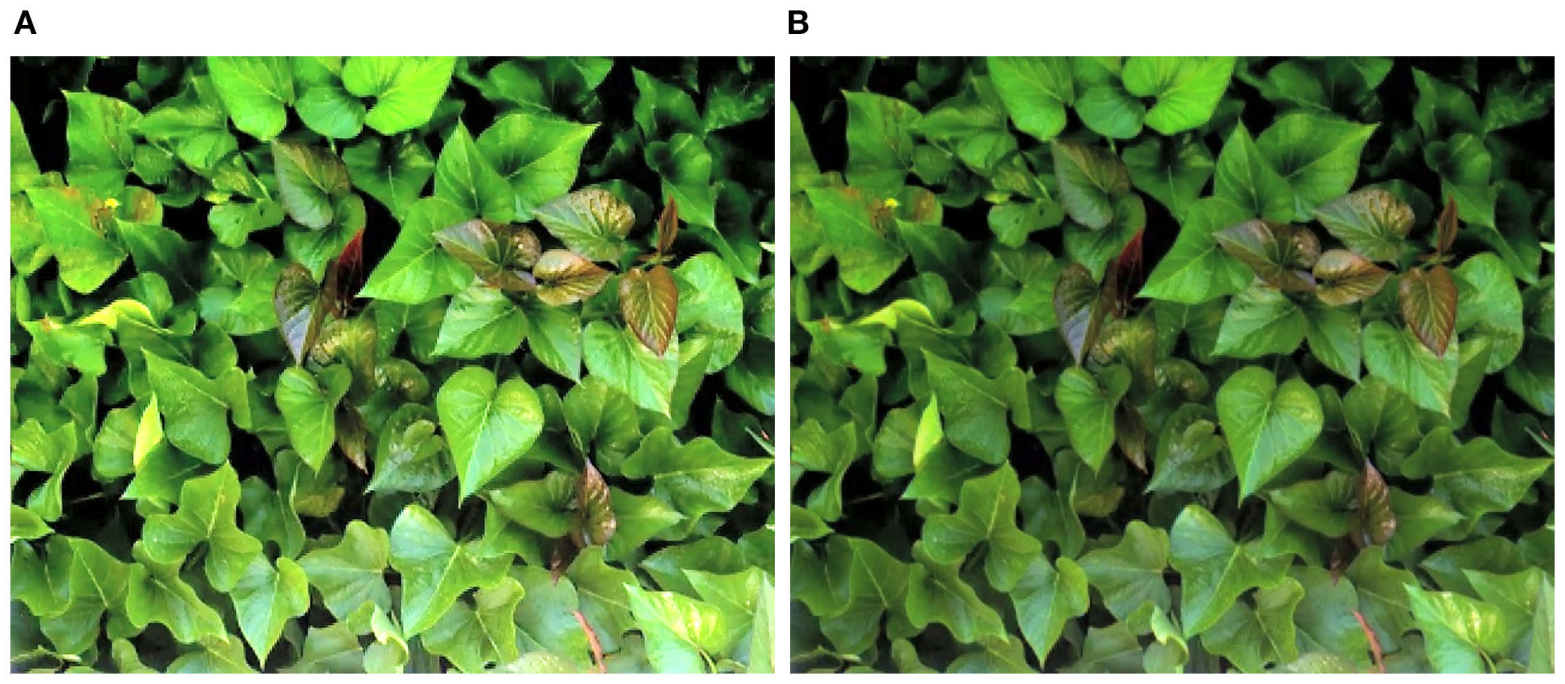
Images processed using the developed calibration program (**(A)** raw image, **(B)** color-calibrated image).

A systematic color calibration protocol was applied to the RGB imagery to ensure consistent and accurate color representation across varying lighting conditions. To conduct color calibration, the color checker was placed within the imaging frame, and the deviation between captured RGB values and the reference values of each color patch was quantitatively calculated. A correction matrix was computed to minimize the differences between each RGB color patch and its corresponding reference value, and this matrix was applied across the entire image to correct for color distortion. To further improve image quality, gamma correction and histogram equalization algorithms were implemented to adjust brightness balance. Lastly, to improve the image analysis accuracy, a foreground-background separation algorithm was incorporated. As a result, high-precision image preprocessing software capable of both color correction and background removal was developed, as illustrated in **Figure 1**.

### 2.4 Outlier removal

A total of 2,399 leaf temperature values and corresponding thermal images were obtained during the study period (June: 489, July: 1,095, August: 815). To improve the quality of the leaf temperature dataset, a normal distribution was used, and values outside the ±3σ range from the mean were considered as outliers. As a result, 14 samples during the early growth stage, one during the tuber bulking stage, and eight during the late growth stage were removed and 2,374 samples were used for model development. thermal images were manually filtered based on the following criteria: (i) if leaf regions were not clearly distinguishable, (ii) if the image was out of focus, or (iii) if non-crop objects or human body parts were visible in the frame. After applying these criteria, a total of 632 thermal images were retained. The same exclusion criteria applied to the thermal images were also used for the RGB images, yielding a final set of 452 valid RGB images.

### 2.5 CWSI acquisition

To calculate the index using the conventional CWSI formula, it is necessary to obtain the theoretically defined wet (T_wet_) and dry (T_dry_) leaf temperatures (Idso et al., 1981). However, in practical field conditions, it is often difficult to directly measure or accurately derive these reference values due to environmental variability and technical limitations (Katimbo et al., 2022; Zhang et al., 2025). This study therefore redefined the conventional CWSI formula based on field-measurable variables to quantitatively assess crop water stress.

To accomplish this, a modified empirical approach using a fixed T_dry_ and measurable environmental variables was proposed. Leaf temperature (T_c_) was measured in real time using a TRI camera and soil moisture content was obtained using a portable soil moisture sensor. Air temperature (T_a_) and relative humidity (RH) were retrieved from the local meteorological administration. Based on these field-acquired data, the CWSI was computed through the following procedure.

Equation 1 estimates the water stress correction coefficient (δ), which quantitatively reflects the crop’s water stress level based on RH and soil moisture conditions. δ was introduced in this study to incorporate water-related physiological stress into the calculation, representing the degree of water deficiency relative to optimal growth conditions. It was calculated using soil moisture content and RH and was formulated as the sum of a base value of 2, an adjustment term (0.1 × |soil moisture − 30|), and a transpiration-related term (2 × (1 − RH/100)). This formulation reflects the physiological response in which the transpiration potential decreases under high RH and water stress intensifies under low soil moisture. To prevent excessive correction, δ was capped at a maximum value of 6 (δ ≤ 6). With this approach the impact of water conditions on plant growth could be more accurately represented.


(1) $$\text{δ}=2+0.1\times |\text{Soil moisture}-30|+2\;\times (1-\frac{\text{RH}}{100})\;(\text{δ}\leq 6)$$

Subsequently, in Equation 2, T_wet_ was estimated by subtracting δ from T_a_, allowing the ideal wet leaf temperature to be indirectly estimated based on the actual air temperature. T_dry_ was fixed at 34 °C, the highest observed leaf temperature in the experimental plots.


(2) $${\text{T}}_{\text{wet }}={\text{T}}_{\text{a}}-\text{ δ}$$

Finally, as shown in Equation 3, the CWSI was calculated using the formula (T_c_ − T_wet_)/(T_dry_ − T_wet_). This formula accounts for both elevated and reduced leaf temperatures as indicators of water stress. Additionally, the CWSI value was constrained to a minimum of 0.05 to minimize distortion due to potential sensor error.


(3) $$\;CWSI=\;\frac{{\lceil \text{T}}_{\text{c}}\;-{\text{ T}}_{\text{wet}}\rceil }{{\text{T}}_{\text{dry}}\;-{\text{ T}}_{\text{wet}}}\;\;\;(0.05\leq CWSI\leq 1)$$

### 2.6 Variable selection for ML model development

In this study, to develop machine learning models for classifying water stress in sweet potato, the most important variables were selected from among the growth indicators presented in Section 2.1, the leaf temperature obtained from thermal image, and the CWSI. To address this, a RF algorithm was used. RF is an ensemble model based on multiple decision trees and is well-suited for variable importance analysis, as it repeatedly uses key variables to construct tree splits and objectively evaluates the degree to which the model depends on each variable (Alduailij et al., 2022; Cao et al., 2020).

In addition, key growth indicators were selected for each growth stage: early growth (June), tuber bulking (July), and late growth (August). This allowed the identification of growth stage-specific factors that influence crop growth and provided data to analyze seasonal or monthly patterns. Furthermore, understanding which variables are most important at each growth stage provides insight into the environmental factors that should be monitored. Ultimately, reducing unnecessary variables improves model performance. Therefore, this approach enabled the extraction of critical growth indicators to support water management decisions.

As shown in **Table 1**, a total of four growth indicators with the highest contribution scores were selected using RF. For the early growth stage, the selected variables were leaf temperature, CWSI, stem length, and CF. For the tuber bulking stage, the most influential indicators were CWSI, SPAD, leaf temperature, and NDVI. Lastly, for the late growth stage, the selected variables were CWSI, stem length, leaf temperature, and CF. Through this analysis, the most influential variables in terms of crop growth and yield prediction were identified, establishing a foundation for optimizing model performance.

**Table 1:** Random Forest-based feature importance across sweet potato growth indicator.

Feature	Early growth stage importance	Tuber enlargement stage importance	Late growth stage importance
Leaf Temperature	0.246	0.161	0.185
CWSI	0.177	0.271	0.265
Stem Length	0.174	0.122	0.178
CF	0.164	0.125	0.089
NDVI	0.139	0.152	0.103
SPAD	0.101	0.172	0.164

### 2.7 ML model development

In this study, five ML models were employed: LR, RF, KNN, MLP, and SVM. LR is a linear classification model that predicts the probability of water stress occurring. It is computationally efficient, interpretable, and well-suited for datasets where class separation is relatively clear, allowing an intuitive understanding of the influence of different variables (Sharma et al., 2021).

RF is an ensemble model that predicts water stress levels by combining multiple decision trees. It exhibits robust performance across various environments and effectively learns complex relationships among variables (Yamparla et al., 2022). KNN classifies new instances by comparing their similarity to the k nearest neighbors in the training set. As it directly reflects data patterns, it is flexible in adapting to data variability (Kumar et al., 2023). MLP is a multi-layer neural network capable of learning nonlinear water stress patterns. It combines diverse features to achieve high representational capacity and can effectively model complex relationships (Bazrafshan et al., 2022). SVM is a powerful classification algorithm that separates water stress levels using hyperplanes. Even when data are not linearly separable, it can learn complex patterns using kernel functions (Behmann et al., 2015; Kok et al., 2021).

Based on the characteristics of each model and the integrated dataset selected via RF, training was conducted separately for each growth stage. A total of 300 integrated data samples consisting of 50 samples per stage were used. The data were stratified according to five water stress levels: SD, D, O, W, and SW, with a ratio of 1:1:2:1:1. The proportion of O-level samples was intentionally doubled to avoid environmental bias. During the experiment, sweet potatoes were cultivated in two greenhouse units—one under D conditions and the other under W conditions. To balance the influence of environmental conditions, O-condition samples were collected from both units. The dataset was split into 80% for training and 20% for testing. Model performance was evaluated based on classification accuracy.

### 2.8 Development of a TRI-RGB fusion-based water stress classifier using a CNN–ViT model

In this study, a CNN-based ViT model was employed. The CNN enables automatic extraction and learning of image features and thus provides faster and more accurate classification compared to manual methods (Oikonomidis et al., 2023). Therefore, it was deemed suitable for classifying TRI and RGB images. By integrating ViT, which can effectively capture general patterns and long-range dependencies that CNN alone might miss, the model was enhanced to more precisely reflect the complex visual characteristics of the crop environment (Lehouel et al., 2024). This architecture strengthened the interaction between features in multimodal images composed of RGB and TRI inputs and contributed to improved classification performance (Lee et al., 2023).

**Figure 2:**
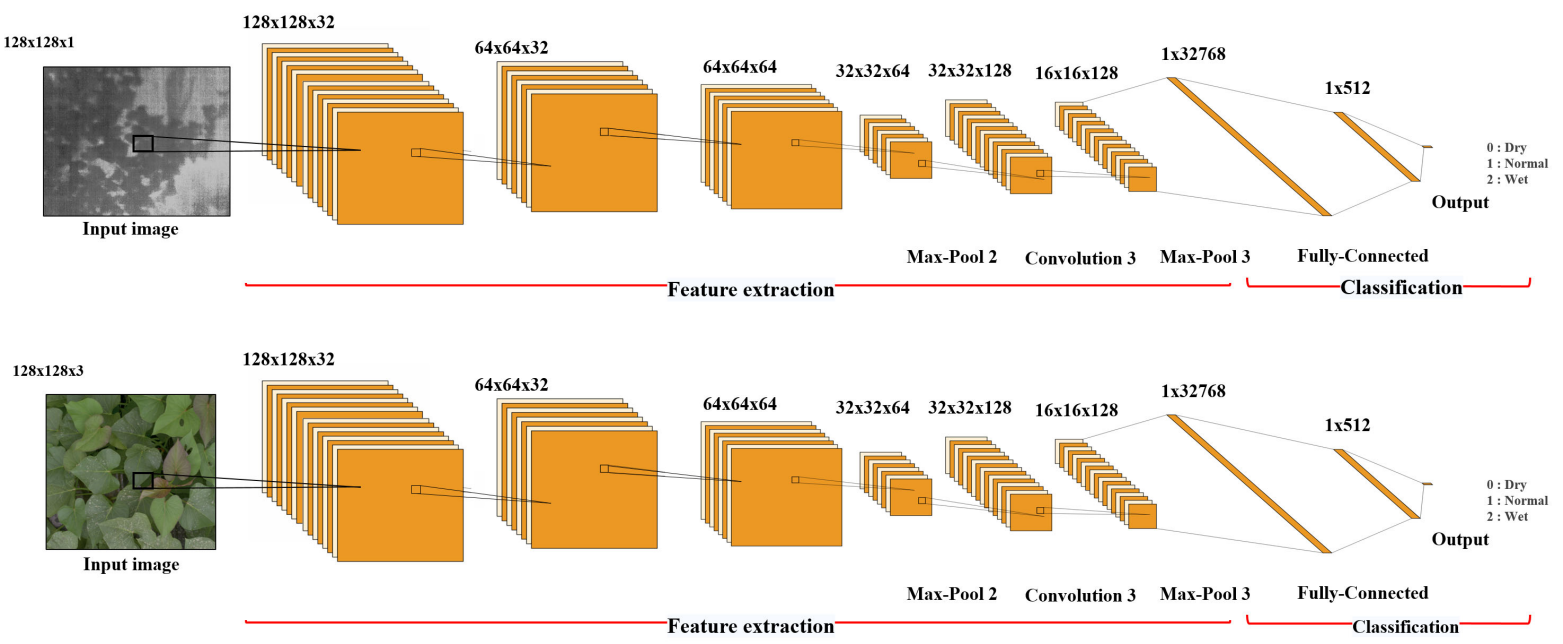
Architecture of the developed CNN-based ViT model.

A total of 904 images, comprising 452 thermal image and 452 RGB images, were used. The dataset was composed of SD (156), D (150), O (150), W (137), and SW (146) images. The data were split into 80% for training and 20% for testing. To ensure stable model performance, k-fold cross-validation was applied. As shown in **Figure 2**, all input images were resized to 128×128 pixels and normalized to the [0, 1] range by dividing the pixel values by 255. RGB images were loaded in “RGB” mode and thermal images in “grayscale” mode, with preprocessing tailored to each sensor type.

For thermal feature maps extracted by the CNN, the mean value was calculated across spatial and batch dimensions to assess the importance of each channel. Only channels with an average activation greater than 0.1 were retained, and the resulting channel mask was uniformly applied across all thermal features. This filtering reduced the number of thermal channels and thereby enhanced input efficiency. Feature maps from the RGB and TRI CNNs were converted into vectors via Global Average Pooling and then concatenated along the channel axis to create a fused feature vector. This fusion vector was used as the input for the final classifier.

The proposed model is a CNN-based ViT that extracts local features from RGB and thermal inputs via separate CNNs; it then combines them into a unified feature vector, which is passed to a Transformer block. The Transformer learns global patterns from this vector and predicts one of the three water stress levels via the final classification layer. The model includes approximately 220,000 trainable parameters and is designed to leverage CNN’s local feature extraction and ViT’s global pattern learning to achieve high classification accuracy with complex multimodal inputs. The training configuration of the CNN-based ViT model is summarized in **Table 2**. Key hyperparameters such as learning rate, batch size, and number of epochs were determined based on prior experiments to achieve optimal performance.

**Table 2:** Training configuration of the CNN-based ViT model.

Hyperparameter	Value
Optimizer	Adam
Learning Rate	Default setting (0.001)
Loss Function	Sparse Categorical Crossentropy
Epochs	Up to 100
Early Stopping	Patience = 10, based on validation loss
Batch Size	64
Validation Strategy	5-fold Stratified K-Fold Cross-Validation
Evaluation Metrics	Accuracy, Confusion Matrix, and Classification Report

### 2.9 Sweet potato water monitor system

GUI is designed to enable users to interact intuitively with a computer through visual elements such as buttons, menus, and images, without the need to input complex commands (Mohammad, 2021). The sweet potato water monitor system was developed using Tkinter, OpenCV, and Pillow to classify the water stress level of sweet potato crops based on RGB and thermal images and to provide appropriate prescriptions accordingly.

**Figure 3:**
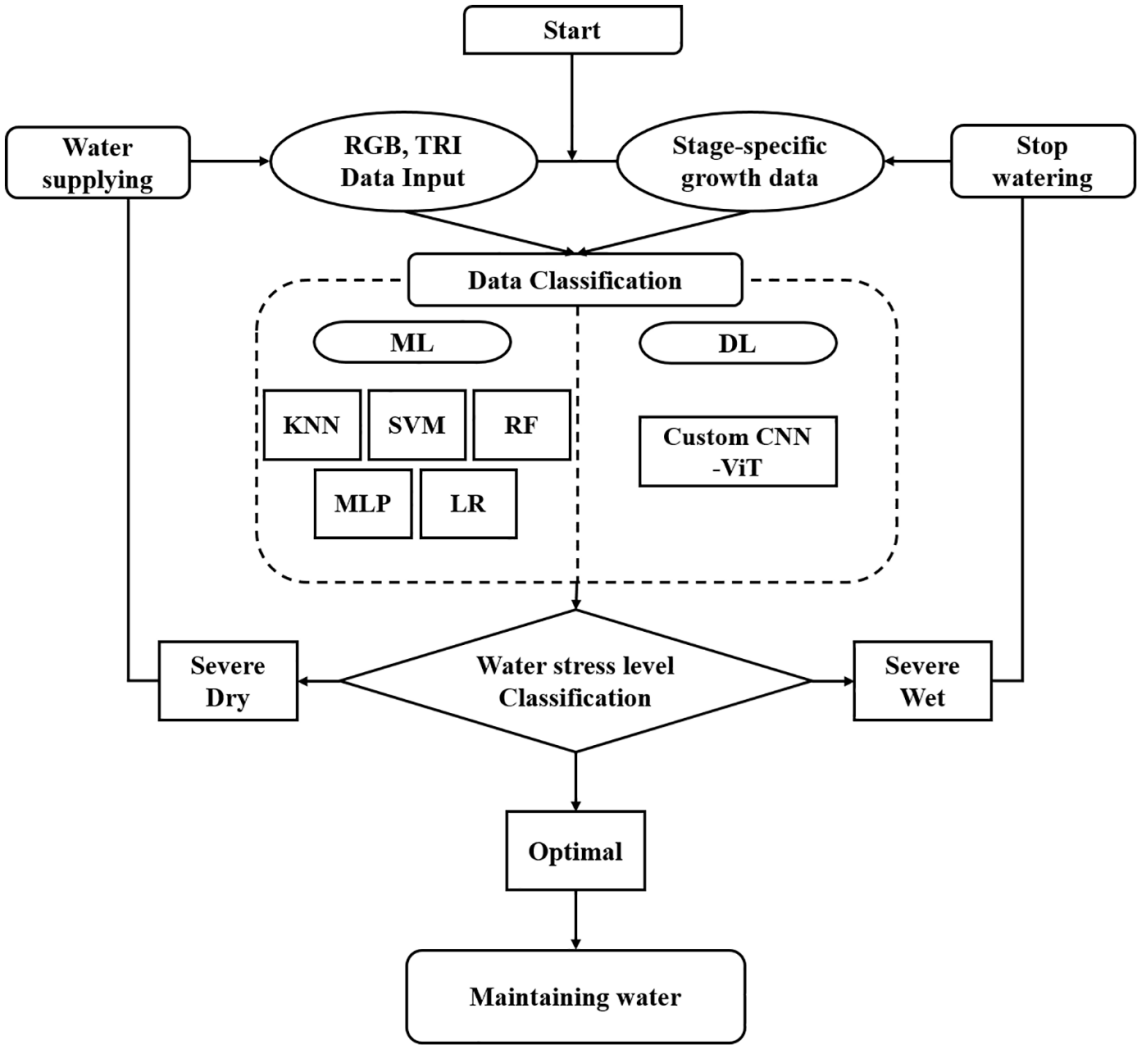
Workflow of the developed sweet potato water monitor system.

Tkinter was employed to implement key interface components, including buttons, labels, and image display areas, while OpenCV and Pillow were used for image loading, preprocessing, and visual output. This design allows users to easily import images and intuitively check the predicted water stress levels along with corresponding management recommendations. **Figure 3** illustrates the step-by-step workflow of the developed GUI software, including the overall implementation process and functional interconnections from image input and model inference to displaying results, gradient-weighted class activation mapping (Grad-CAM)-based visualization, and sensor data processing. This flowchart provides practical insight into the logical structure and operational sequence of the software.

### 2.10 Model evaluation and experimental setup

The performance of the developed models was evaluated using the test dataset. The evaluation was based on a confusion matrix, which compares the model predictions with the actual ground truth and consists of true positives (TP), true negatives (TN), false positives (FP), and false negatives (FN). Based on the confusion matrix, key performance metrics—accuracy, recall, precision, and F1-score—were calculated to quantitatively assess the classification performance of each model. The formulas used for these metrics are provided in **Equations 4**–**7**.


(4) $$\text{Precision}=\frac{\text{TP}}{\text{TP}+\text{FP}}$$

(5) $$\text{Recall}(\text{Sensitivity})=\frac{\text{TP}}{\text{TP}+\text{FN}}$$

(6) $$\text{F}1\text{ Score}=2\times \frac{\text{Precision}\times \text{Recall}}{\text{Precesion}+\text{Recall}}$$

(7) $$\text{Accuracy}=\frac{\text{TN}+\text{TP}}{\text{TN}+\text{TP}+\text{FP}+\text{FN}}$$

A 5-fold cross-validation procedure was employed to account for the limited dataset size. To mitigate evaluation bias under class imbalance, a stratified scheme was applied so that each fold preserved the original class distribution. The data were shuffled at each split, and a fixed random seed (e.g., random_state = 42) was used to ensure reproducibility. In each fold, approximately 80% of the data were allocated for training and 20% for validation. All preprocessing steps (e.g., feature scaling) were fitted on the training subset only and subsequently applied to the corresponding validation subset to prevent data leakage. For fair comparison, the same fold partitions were consistently applied across all models. Performance metrics, including accuracy, macro/weighted F1-score, precision, and recall, were reported as mean ± standard deviation across the five folds.

All experiments were conducted on a Windows operating system using Jupyter Notebook (Anaconda Inc., USA). A GeForce GTX 1060 GPU (NVIDIA, USA) was employed for model training and inference. Python version 3.9.18 (Python Software Foundation, USA) was used as the programming environment and TensorFlow version 2.10.0 (Google Brain Team, USA) served as the deep learning framework.

## 3 Results and discussion

### 3.1 Statistical difference verification using ANOVA

To evaluate the statistical significance of the leaf temperature data after removing outliers, an analysis of variance (ANOVA) was performed. This statistical method is used to compare data groups based on a single independent variable (group or category). In this study, ANOVA was used to determine whether mean leaf temperatures significantly differed among the five water stress levels. As shown in **Table 3**, the analysis resulted in an F-statistic of 2.376 and a p-value of 0.043. Because the p-value is less than 0.05, it indicates that the mean leaf temperature differs significantly across water stress classes. In other words, the leaf temperature of sweet potatoes varies depending on the water treatment level.

**Table 3:** ANOVA analysis of variance for sweet potato leaf temperature classification levels.

Source of variation	Sum of squares	Degrees of freedom	Mean square	F-value	p-value	F-critical
Treatment	382.95	4	95.74	12.57	4.34×10^-2^	2.38
Error	17607.61	2312	7.62			
Total	17990.57	2316				

### 3.2 ML model training results

**Figure 4:**
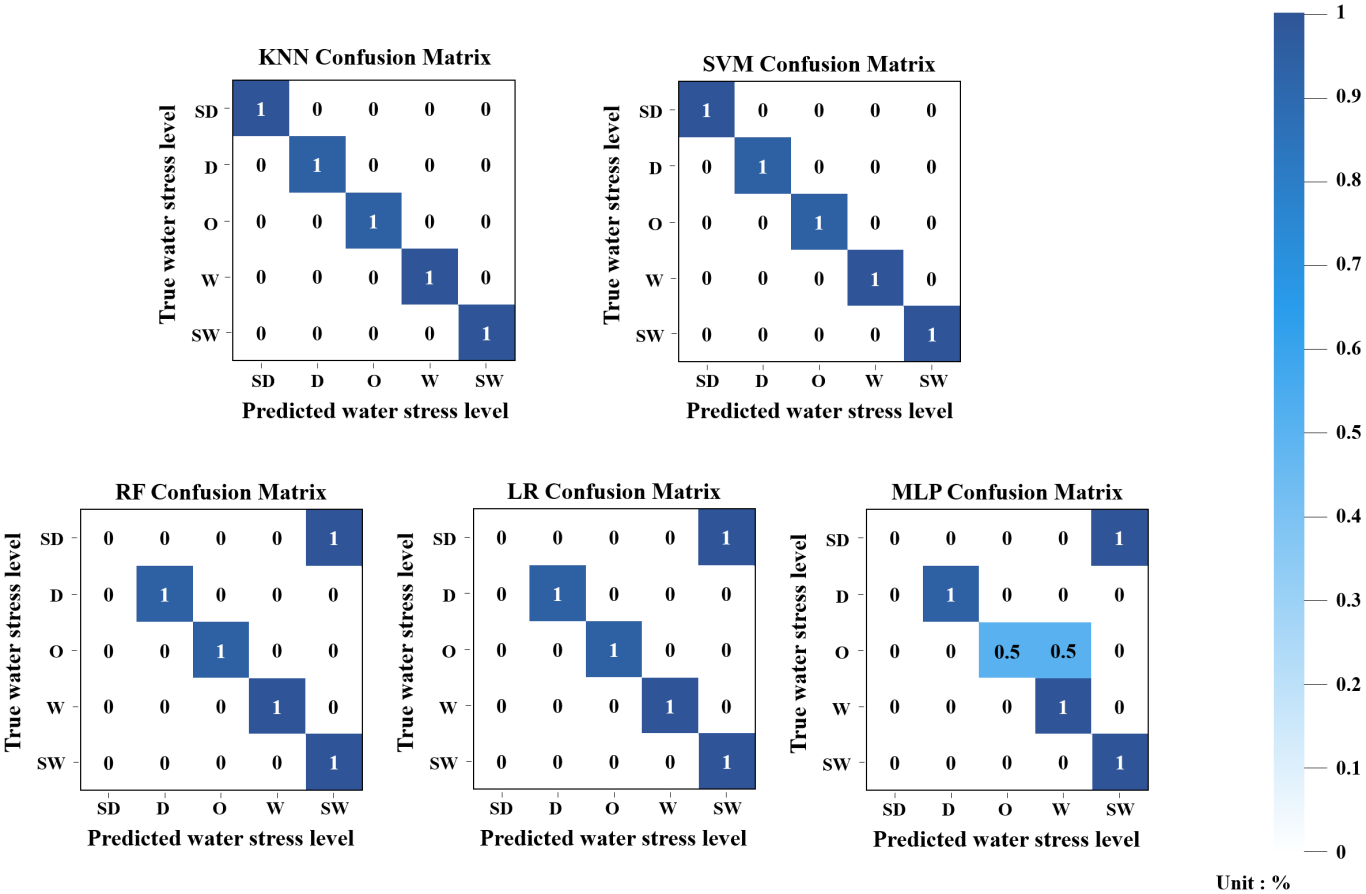
Confusion matrix results of five ML classification models using the June integrated dataset.

The ML models for classifying sweet potato water stress levels were trained separately for each growth stage using five algorithms: LR, RF, KNN, MLP, and SVM. In June, as shown in **Figure 4**, both KNN and SVM achieved perfect classification accuracy (1.0), while RF and LR each recorded a value of 0.84, and MLP showed lower performance at 0.67. In the RF and LR models, samples from the SD class were misclassified as SW, indicating decreased classification performance in distinguishing extreme water levels. MLP also showed similar misclassification patterns, with 50% of O-class samples incorrectly classified as W-class. These results suggest that the models had difficulty distinguishing between certain adjacent classes, which may be attributed to overlapping feature characteristics or an imbalanced data distribution.

**Figure 5:**
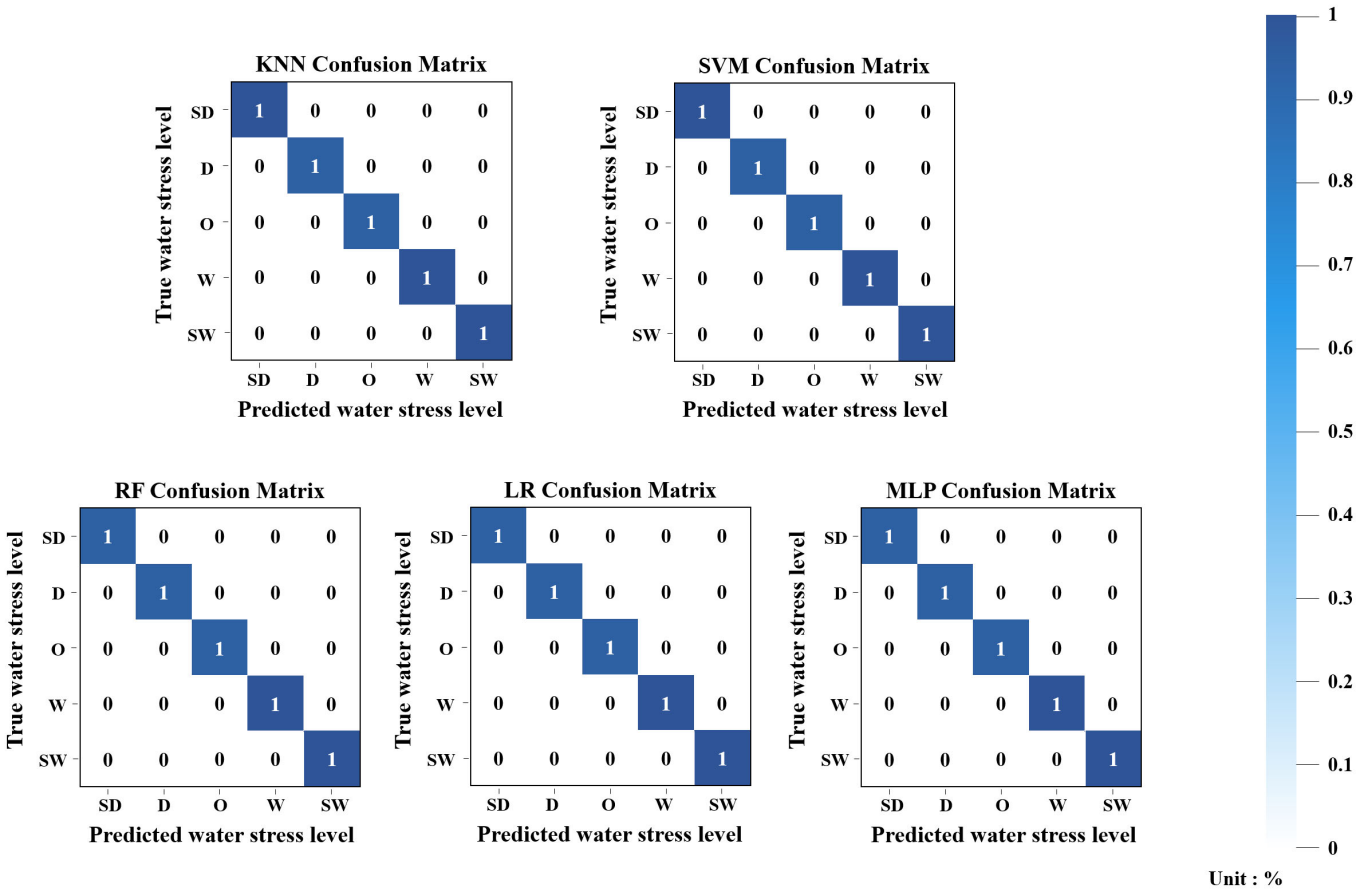
Confusion matrix results of five ML classification models using the July integrated dataset.

In July, all models achieved perfect accuracy (1.0) as shown in **Figure 5**, suggesting they effectively learned distinct features from the test data. Considering that July samples might have had more distinguishable characteristics compared to other months, these results likely reflect strong generalization rather than overfitting.

**Figure 6:**
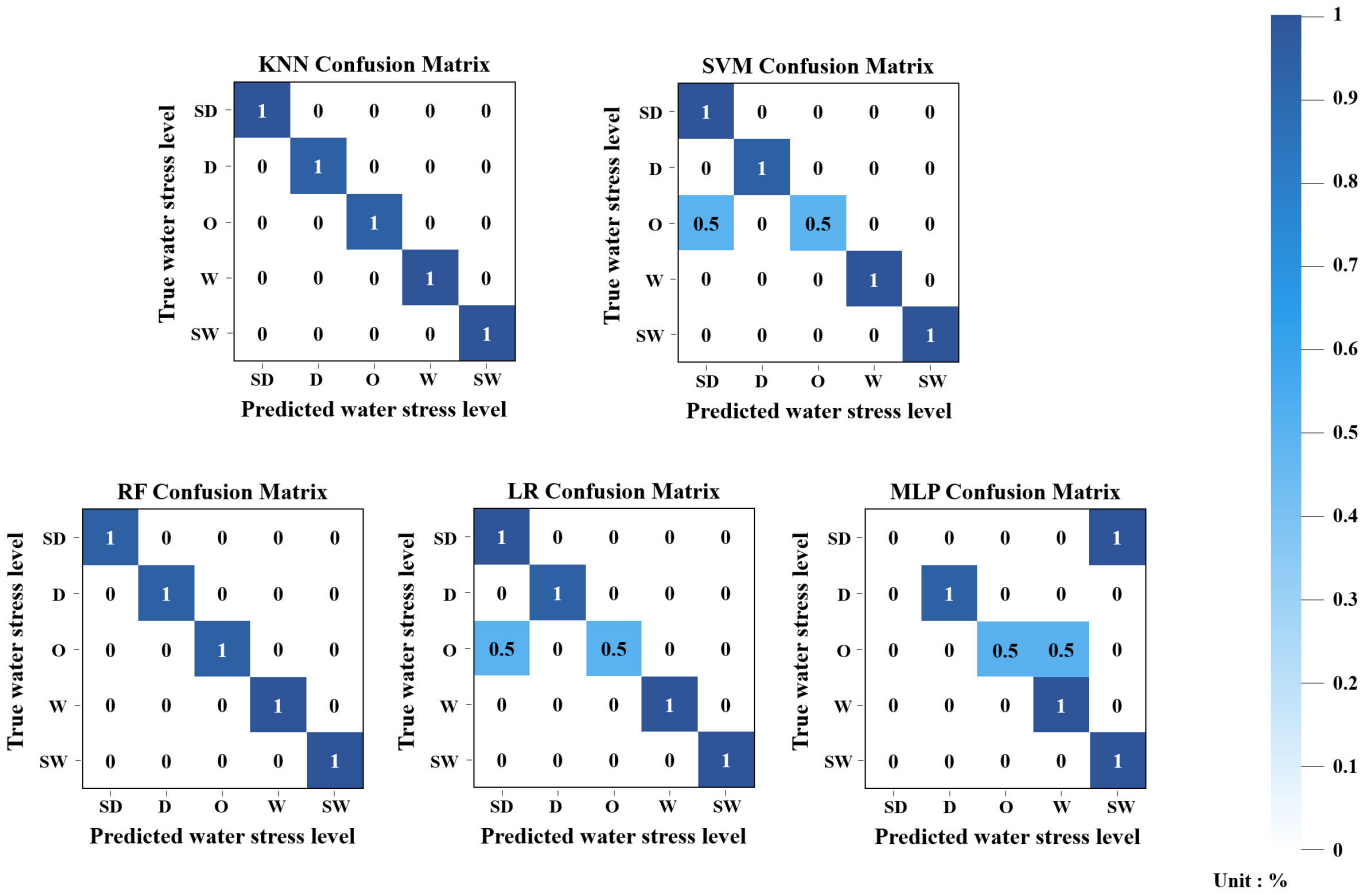
Confusion matrix results of five ML classification models using the August integrated dataset.

In August, according to **Figure 6**, KNN and RF again achieved perfect accuracy (1.0), while SVM, LR, and MLP each showed accuracy of 0.84. All three models misclassified 50% of O-class samples as SD. This implies that these models failed to differentiate O from SD, possibly due to overlapping features or the ambiguous nature of mid-range water levels (O), which may not exhibit clear boundaries compared to the more extreme SD class.

**Figure 7:**
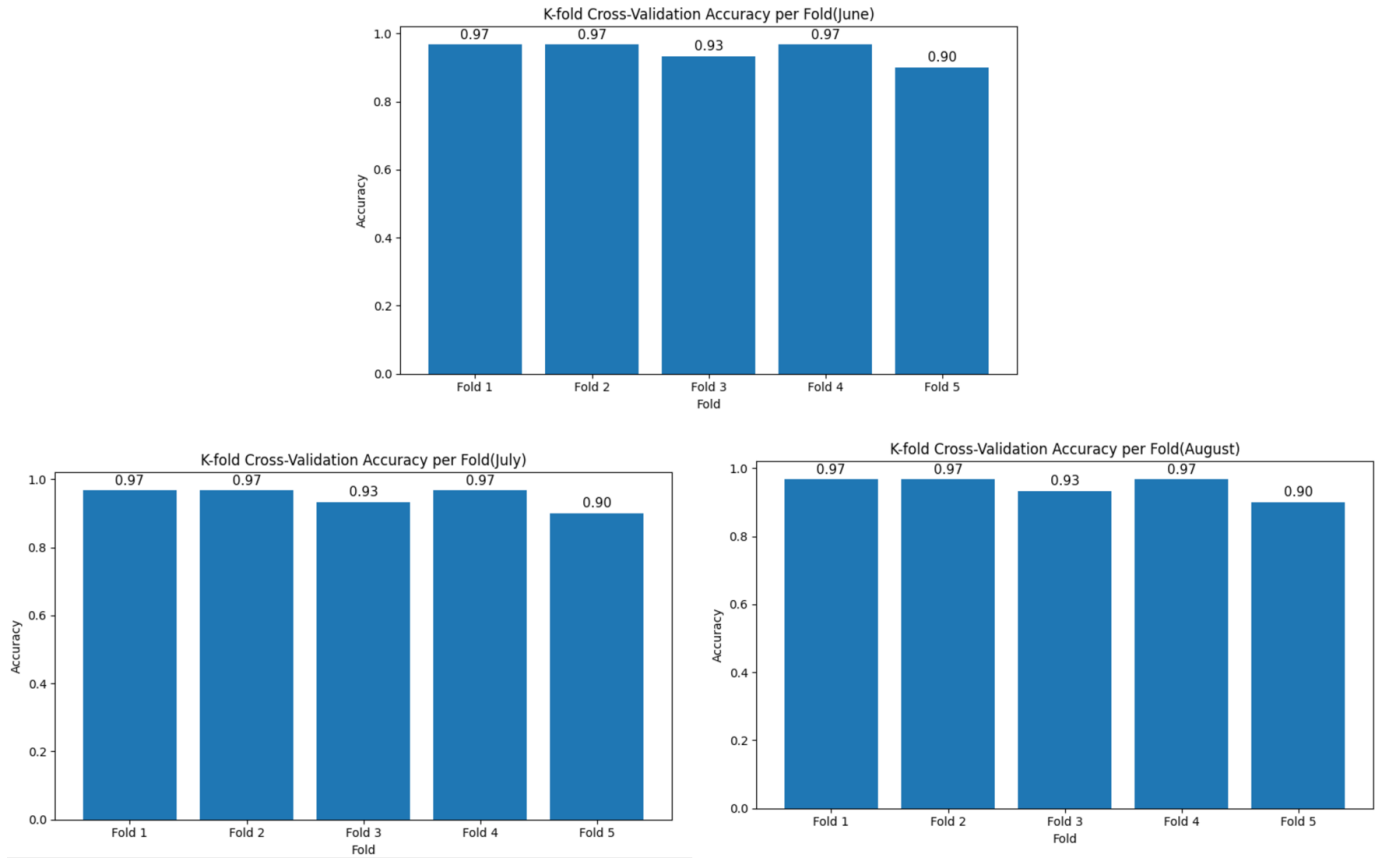
Distribution of 5-fold cross-validation accuracy by month.

Although all models initially achieved perfect classification accuracy on the test sets, this raised concerns about potential overfitting. Therefore, 5-fold cross-validation was performed to evaluate the generalization performance, yielding average validation accuracies of 0.94 in June, 0.96 in July, and 0.94 in August. The distribution of cross-validation accuracies for each month is presented in **Figure 7**. Although these values are slightly lower than the initial test accuracies, the results still demonstrate strong model performance and indicate that the proposed approach maintains robust generalization capability across different growth stages.

### 3.3 Final ML model selection

In the results of the comparative analysis of multiple classification models using integrated data by sweet potato growth stages the KNN model consistently demonstrated the highest classification accuracy across all stages. This outcome can be attributed to the structural characteristics of the KNN algorithm and its synergistic compatibility with the numerical patterns embedded in the integrated dataset. KNN is a non-parametric algorithm that does not construct an explicit model during training. Instead, it performs classification by calculating distance-based similarity between input data and training samples at prediction time (Zhang, 2022; Uddin et al., 2022).

This is particularly effective when dealing with well-structured, quantitatively distinctive datasets such as the integrated data used in this study. KNN effectively captured the structure of each growth stage and formed clear decision boundaries among similar samples. Consequently, it successfully distinguished the numerical band information associated with sweet potato growth characteristics.

Moreover, KNN does not require assumptions about the data distribution, and thus is especially advantageous when processing non-linear or multivariate traits of growth indicators that vary by growth stage (Bansal et al., 2022). When the color characteristics of sweet potato leaves maintain a consistent similarity-based structure in high-dimensional space across stages, KNN can leverage this to outperform other models in classification performance (Sagana et al., 2022). In conclusion, the structural flexibility and distance-based classification mechanism of KNN align well with the numerical properties of sweet potato integrated data. This is considered a key factor in explaining the consistent superiority of the KNN model’s classification performance across all growth stages in this study.

### 3.4 DL model training results

**Figure 8:**
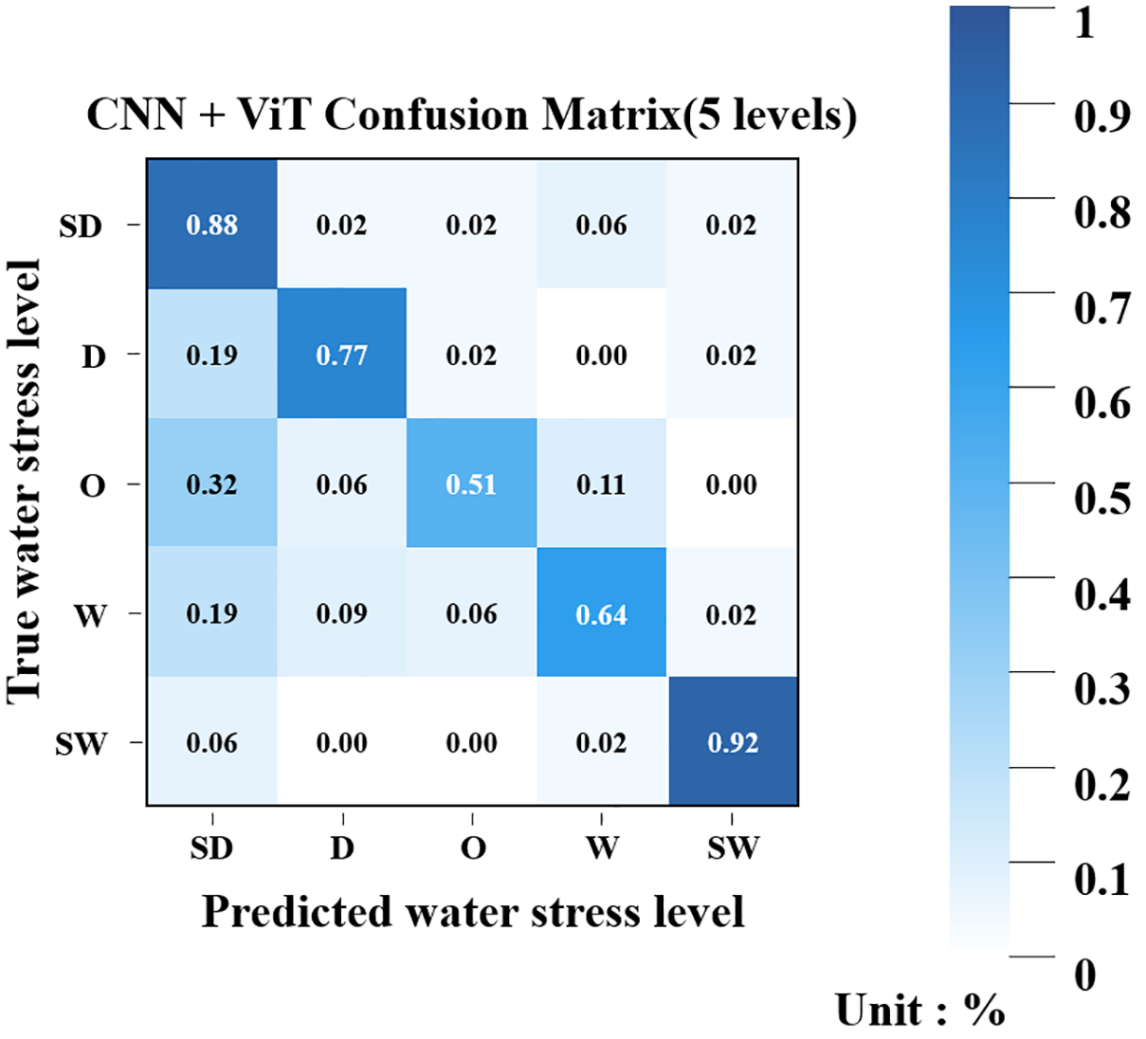
Confusion matrix of the CNN+ViT model for five water stress levels.

In this study, a CNN-based ViT classification model was employed to classify sweet potato water stress levels using a fused image composed of thermal images and RGB images. Unlike the machine learning models based on integrated data, the fused image classification model did not divide the water stress levels into five categories. Instead, it consolidated the extreme water conditions (SD and SW) and classified them into three categories: D, O, and W. As shown in **Figure 8**, dividing the water stress levels into five intervals resulted in reduced classification accuracy (0.75) and elevated loss (0.7)—this suggests that the model struggled to differentiate between finer stress categories. Although extreme water conditions were relatively well identified through image-based observations, the visual features between the dry and wet conditions was ambiguous. This makes it difficult for the model to differentiate between the two classes, which led to a decrease in classification accuracy. Therefore, the water stress levels were restructured based on the degree to which they could be identified through image information alone, with the aim of establishing a more reliable classification framework.

**Figure 9:**
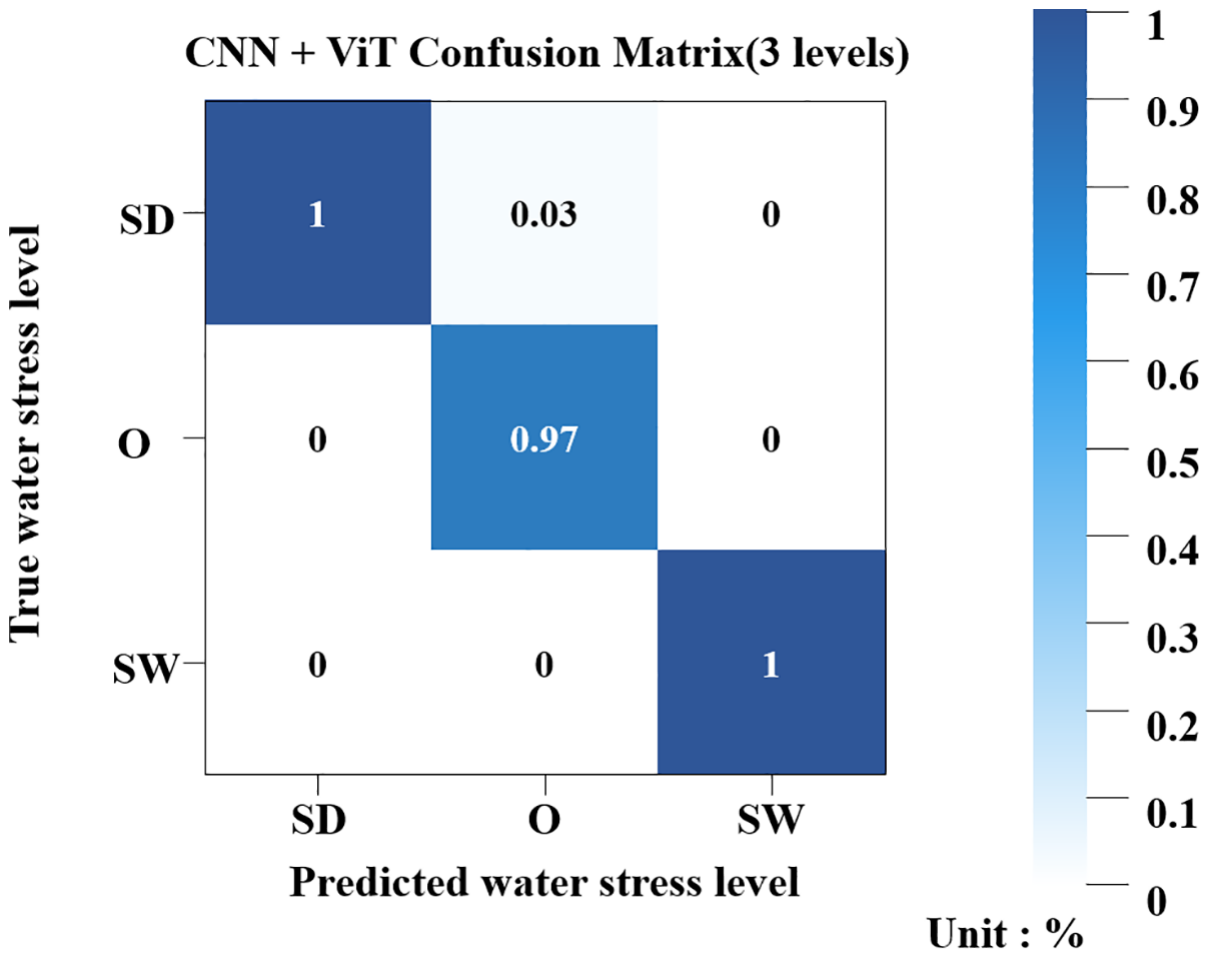
Confusion matrix of the CNN+ViT model for three water stress levels.

As a result of training the simplified stress-level classification model, the accuracy reached a high value of 0.92. **Figure 9** presents confusion matrices of the TRI and RGB classification models. For the three water stress levels (SD, O, SW), the model exhibited generally high classification accuracy, achieving 100% accuracy for both the SD and SW classes. The O class showed slightly lower performance with an accuracy of 97%, with 3% of its samples misclassified as SD. The application of 5-fold cross-validation resulted in an average accuracy of 0.91.

### 3.5 Development of the sweet potato water monitor system

**Figure 10:**
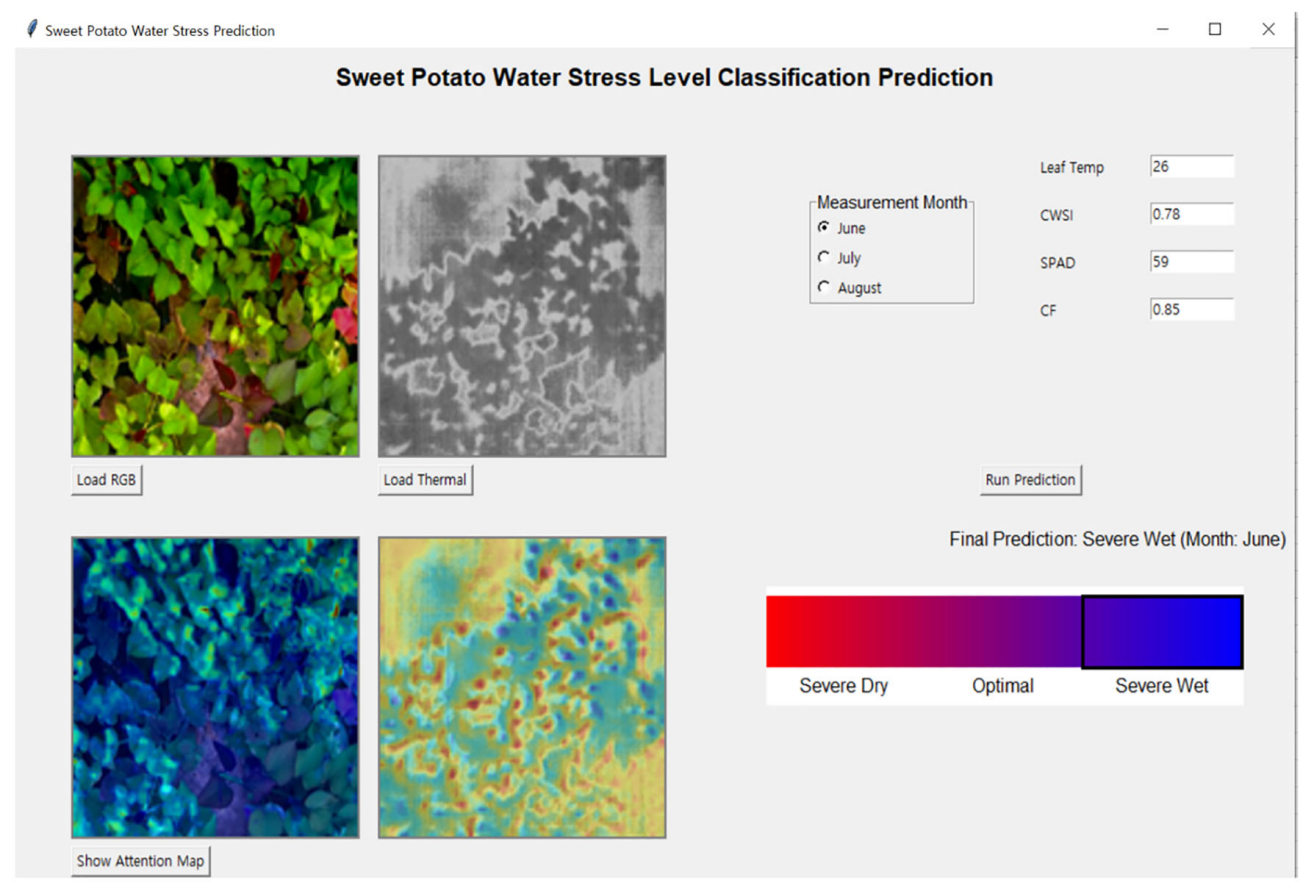
The developed GUI software.

In this study, a sweet potato water monitor system was developed by integrating the KNN model with the CNN-based ViT model to effectively classify the water stress levels of sweet potato crops (**Figure 10**). The developed system is a deep learning-based RGB–thermal CNN+ViT prediction framework designed to visualize and predict water stress levels in sweet potato crops, consisting of four main interface components. It requires only modest computational resources (≈30.5M parameters; ~122 MB in fp32) and operates in near real time on a CPU, with GPU acceleration further reducing latency. Moreover, Grad-CAM explanations are computed on-the-fly with moderate overhead, enabling practical field deployment with consumer-grade hardware.

The top-left section of the GUI is the input image area, where users can load RGB and thermal images separately. The ‘Load RGB’ button on the left allows users to import RGB images that capture the visual characteristics of sweet potato leaves, while the ‘Load Thermal’ button on the right inputs thermal images reflecting the leaf temperature distribution. These two image types are utilized independently in the CNN-based feature extraction process of the model to analyze visual information.

The top-right section is the sensor-based input area. This area includes radio buttons for selecting the growth period (June, July, or August) and allows manual input of numerical growth indicators corresponding to each period. These input values are fed into the KNN-based auxiliary classifier, which forms a fused prediction architecture in conjunction with the deep learning inference.

The bottom-right section serves as the output area for displaying the prediction results. Upon clicking the ‘Run Prediction’ button, the prediction outputs from the CNN+ViT and KNN models are averaged to produce a final water stress level. The result is presented as one of three classes: Severe Dry, Optimal, or Severe Wet. A gradient-colored bar beneath the prediction output visually highlights the predicted stress level, providing intuitive feedback to the user.

The bottom-left section corresponds to the attention map visualization area. When the ‘Show Attention Map’ button is clicked, the GUI displays visual explanations generated by Grad-CAM based on Explainable AI (XAI). The left side shows the attention regions derived from the RGB image, and the right side displays those from the thermal image. This allows users to intuitively identify the regions the model focused on during decision-making and enhances interpretability and trust in the model.

This sweet potato water monitor system is designed to predict the water stress condition of crops based on both image data and growth indicators, while visually explaining the decision-making process of the deep learning model.

**Figure 11:**
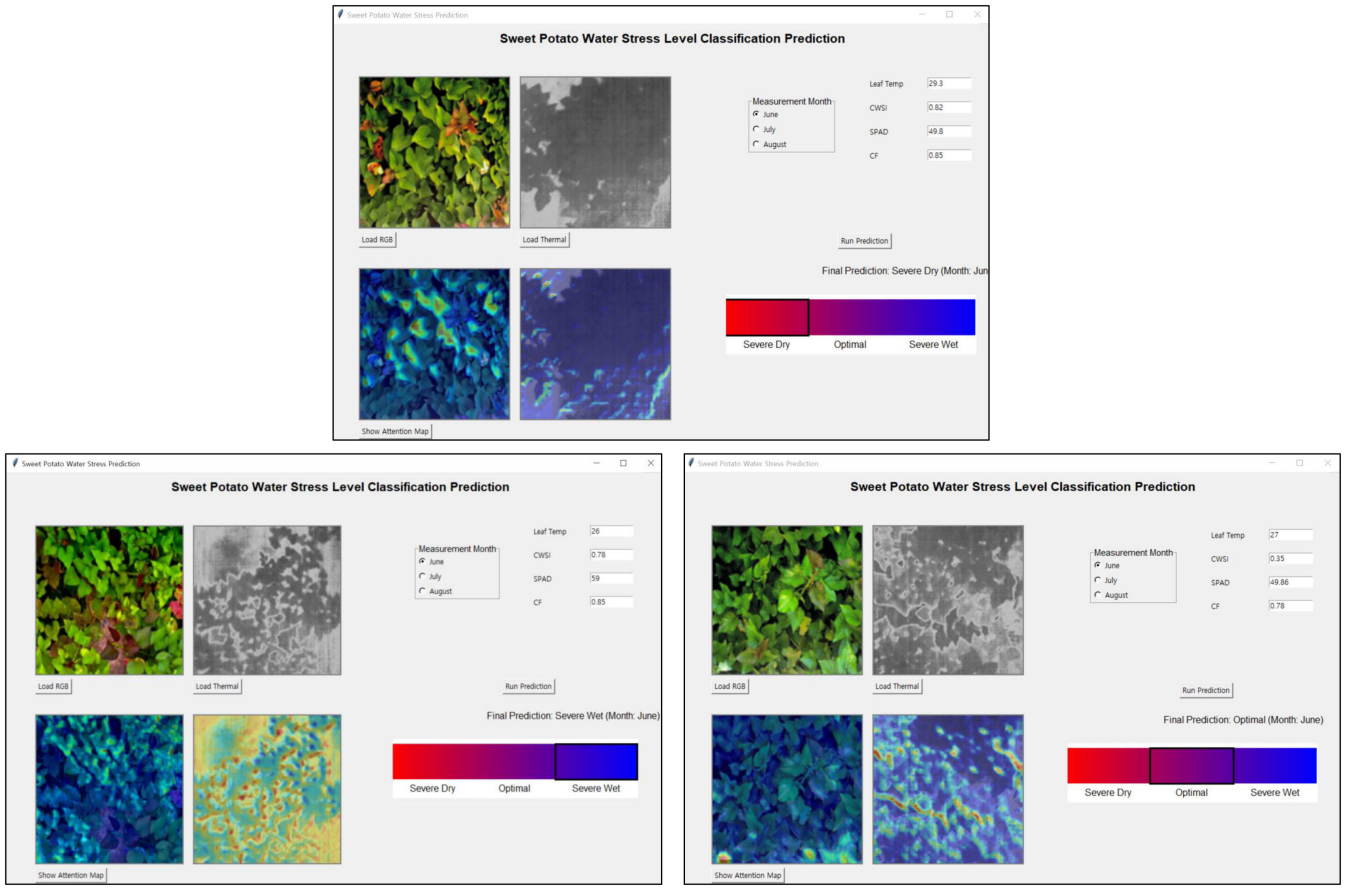
Representative images showing the classification of crops into three water stress levels: SD, O, and SW.

**Figure 11** illustrates representative images corresponding to the classification of crops into three distinct water stress levels: SD, O, and SW.

### 3.6 Discussion

#### 3.6.1 Model evaluation

This study compared and analyzed the classification characteristics and performance of two approaches for sweet potato water stress classification: a quantitative ML model based on growth indicators and an image-based DL model using fused RGB and thermal images. Specifically, a KNN model based on integrated data including SPAD, CWSI, and leaf temperature was combined with a ViT-based CNN architecture applied to image data to establish a complementary prediction framework.

The ML-based KNN model demonstrated stable classification performance, particularly for moderate water stress levels, by leveraging quantitative growth indicators. In contrast, the DL-based model exhibited high sensitivity in extreme stress conditions. Notably, the KNN model achieved the highest classification accuracy across all growth stages, which can be attributed to its structural compatibility with quantitative data.

The experimental results revealed that when the water stress levels were classified into five categories, the model achieved a relatively low accuracy of 0.75 with an elevated loss of 0.7. By contrast, simplifying the stress levels into three categories (SD, O, SW) substantially improved classification accuracy to 0.92, with 5-fold cross-validation confirming robust performance at an average accuracy of 0.91. The confusion matrices further showed that both the SD and SW classes were identified with 100% accuracy, while the O class reached 97%, with only 3% of the samples misclassified as SD.

This restructuring was motivated not only by empirical performance improvements but also by practical considerations. In agricultural practice, irrigation and fertilization decisions are generally aligned with broad soil moisture conditions such as deficit, optimal, and excess, rather than highly granular categories. Thus, a three-level classification scheme is more consistent with real-world management practices and easier to implement on small-scale farms that lack automated control systems.

From a modelling perspective, thermal and RGB imagery were effective for detecting extreme stress levels but provided limited cues for differentiating intermediate conditions. Consolidating the categories allowed the CNN-based ViT model to focus on clearer visual signals, which enhanced both robustness and generalizability. Furthermore, the simplified classification outputs can be directly translated into actionable recommendations such as “initiate irrigation,” “maintain current conditions,” or “reduce watering,” thereby bridging the gap between advanced image-based analytics and practical decision support in agriculture. The primary distinction of this study is that it provides an integrated analysis of sweet potato water stress by combining image data (RGB and thermal imagery) with growth indicators such as SPAD, CWSI, and leaf temperature. This fusion-based approach offers significant advantages that not only enhance model accuracy but also improve interpretability and practical applicability in real-world agricultural settings.

#### 3.6.2 Previous studies

In contrast with the present study, most previous research has not systematically classified the water stress levels of sweet potato crops or integrated image-based analysis with growth indicators. Existing studies on detecting and classifying water stress have primarily focused on major cereal crops such as rice, maize, sugarcane, and soybean. For example, Kapari et al. (2024) conducted an evaluation of maize water stress and crop growth monitoring in small-scale farms using UAV data. The results indicated that the Random Forest model achieved a coefficient of determination (R²) of 0.85, a root mean square error (RMSE) of 0.05, and a mean absolute error (MAE) of 0.04 (Kapari et al., 2024). de Melo et al. (2022) proposed a method for predicting water stress in sugarcane crops using the deep neural network Inception-ResNet-v2 trained on thermal images. The analysis demonstrated improved performance, achieving accuracy increases of 23%, 17%, and 14% for the AWC levels of 25%, 50%, and 100%, respectively, compared with previous approaches (de Melo et al., 2022). Mokhtar et al. (2023) applied six ML and DL models to predict the irrigation water requirement (IWR) of snap beans using various weather, soil, and crop variables as inputs. The results showed that, under the S7 scenario, the DNN model achieved the best performance with MBE = –0.001, RMSE = 0.055 mm, and NSE = 0.824 (Mokhtar et al., 2023).

In the present study, a systematic classification of sweet potato water stress levels was conducted by incorporating multiple growth indicators along with leaf temperature data. Among the ML models, KNN achieved the highest performance, reaching an accuracy of 1.00 across all growth stages, while maintaining strong results in 5-fold cross-validation (0.94, 0.96, and 0.94). Furthermore, the integration of RGB and thermal images using a CNN+ViT model demonstrated superior performance, achieving an accuracy of 0.97, with a 5-fold cross-validation result of 0.91. These reported results were then compared with the findings of the present study, as summarized in **Table 4**.

**Table 4:** Comparison of previous studies with the present study results.

Study	Crop	Best model	Performance metrics
Kapari et al. (2024)	Maize	RF	R² = 0.85, RMSE = 0.05, MAE = 0.04
Melo et al. (2022)	Sugarcane	Inception-ResNet-v2	Accuracy improved by 23% (AWC 25%), 17% (AWC 50%), 14% (AWC 100%) vs. baseline
Mokhtar et al. (2023)	Snap bean	Deep Neural Network (DNN)	MBE = –0.001, RMSE = 0.055 mm, NSE = 0.824
Present study	Sweet potato	CNN–ViT	Accuracy: 0.91
Present study	Sweet potato	KNN	Accuracy: 0.94-0.96

Previous studies proposed models that quantitatively predicted water stress by combining UAV-derived MSI or HSI with growth indicators (Wang et al., 2024; Dong et al., 2024; Sharma et al., 2025; Brewer et al., 2022). Although these approaches demonstrated a reasonable levels of predictive performance, their applicability in real agricultural settings is limited due to the high cost of equipment and the complexity of data processing and interpretation. Moreover, most existing studies were restricted to specific crop groups, particularly cereals, and often involved limited types of image data and growth indicators. In contrast, root crops such as sweet potato—despite that root enlargement is a key growth indicator—have rarely been analyzed using an integrated approach that combines physiological and image-based data to assess water stress.

Notably, instead of relying solely on RGB images or individual growth indicators, this study implemented a practical and scalable analytical platform that integrates diverse sensor-based quantitative physiological indicators with image data. This multidimensional approach captures sweet potato responses to water stress more comprehensively. Furthermore, this study integrated XAI techniques based on Grad-CAM into the sweet potato water monitor system to provide a visual explanation of prediction outcomes. Users can identify the specific regions of the image that the model focused on during inference, which significantly enhances the model’s transparency and its applicability in real-world agricultural settings. This visualization feature offers practical advantages and offers potential for direct application in crop management.

#### 3.6.3 Limitations of this study

However, several limitations of this study should be acknowledged. First, the image-based classification accuracy for the O class was relatively low and consequently moderate stress conditions were difficult to distinguish. This limitation is primarily attributed to the intrinsic constraint of image data in capturing detailed physiological responses. Second, the dataset exhibited class imbalance due to an insufficient number of samples in some categories, which may have introduced bias in model training. Third, since the study relied on static images and growth indicators, it could not reflect temporal variations in growth or the accumulation patterns of stress over time. Lastly, the absence of a quantitative analysis for sensory and chemical traits such as sweetness, taste, and quality of sweet potato is an area that should be addressed in future work.

#### 3.6.4 Future work

Future work will focus on advancing the developed classification and prediction system into a deployable platform for agricultural use. A fixed-position camera system will periodically capture crop images and automatically transmit them to an online server for processing. Through this infrastructure, users will be able to access prediction results, Grad-CAM visualizations, and sensor data in real time via a web-based application, without requiring additional software installation. Such an automated platform will improve accessibility and provide both quantitative and visual assessments of crop water status. Moreover, the validated algorithmic framework is not limited to sweet potato; developing crop-specific models may enable expansion into a general-purpose diagnostic platform adaptable to diverse cultivation environments.

The system also demonstrates high practical feasibility, as it is more cost-effective than hyperspectral equipment and can be readily utilized by agricultural practitioners. It is being designed with an intuitive user interface, essential data management functions, and integration potential with existing farm management tools. Although the detailed specifications of the web platform and software architecture have not yet been finalized, the design process prioritizes applicability in real-world settings.

In terms of economic impact, the deployment of the proposed system may reduce reliance on costly sensing platforms such as hyperspectral cameras and lower labor requirements for manual monitoring, thereby contributing to reduced operational costs. Furthermore, by providing real-time, automated, and interpretable predictions, the system is expected to enhance water-use efficiency and minimize resource waste, ultimately improving cost-effectiveness for agricultural practitioners.

**Figure 12:**
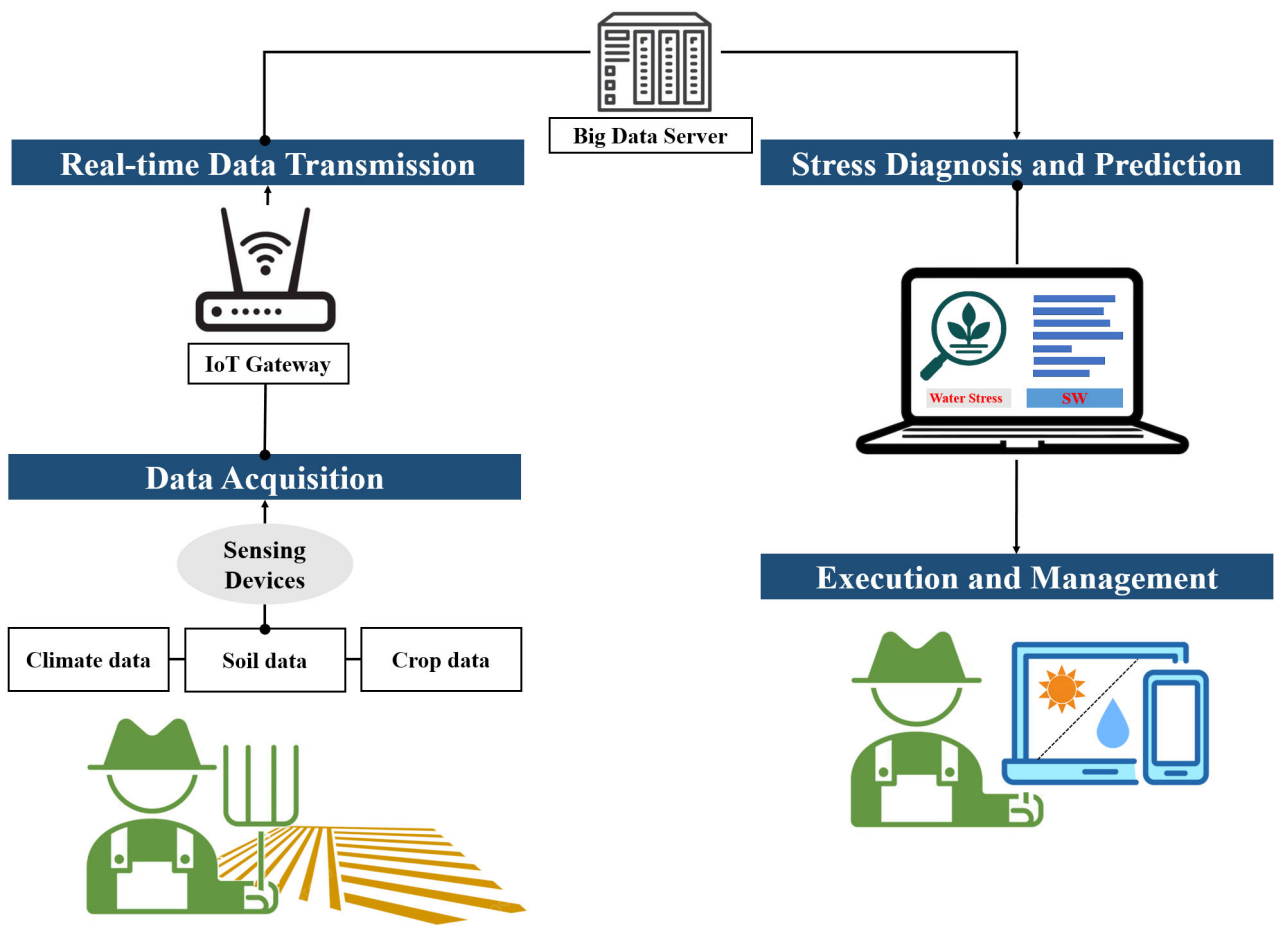
Flowchart of the agricultural management decision-making process.

**Figure 12** illustrates the agricultural management decision-making process. The flow begins with data acquisition (climate, soil, and crop imaging), followed by data transmission and preprocessing, leading to AI-based analysis for water stress diagnosis and prediction. The outputs then inform management decisions such as irrigation, fertilization, and environmental control, completing a cycle that supports efficient agricultural management.

## 4 Conclusion

This study developed and analyzed both an integrated data-based ML model and a ViT-CNN DL model using RGB and thermal image acquired from low-altitude platforms to efficiently classify and predict the water stress levels of sweet potato. Among the ML models, the KNN classifier achieved the highest accuracy across all growth stages. The ViT-CNN model also demonstrated strong performance, particularly through the application of a class simplification strategy that improved prediction accuracy. Notably, restructuring the water stress levels from five to three classes helped overcome the limitations of image-based classification, reducing potential misclassification and enhancing reliability for real-world applications.

To improve practicality under open-field cultivation conditions, certain meteorological variables required for calculating the CWSI were replaced with field-observable elements. A redefinition of the index formula was proposed based on these replacements to provide a more realistic computation of CWSI and increase the utility of TRI-based growth indicators. This approach allows a CWSI-based quantitative analysis even in limited sensor environments.

In summary, the developed sweet potato water monitor system—integrating XAI visualization via Grad-CAM and a user interface for sensor-based input—was designed to enable users to intuitively assess crop water status and obtain appropriate recommendations. This configuration offers both practicality and scalability, supporting its application in real agricultural settings.

Future work will be carried out to build a fixed camera-based imaging system, wherein data will be collected and processed through an online infrastructure and delivered via a web-based application platform. This extension will enable real-time monitoring and facilitate the application of the system to other crops, thereby contributing to the advancement of smart agriculture technologies. In addition, further validation will be conducted using multiple sweet potato cultivars, including ‘Jinyulmi’, ‘Shinyulmi’, and ‘Pungwonmi’, to assess the system’s generalizability. Temporal analysis will also be incorporated to capture the progression of water stress over time, providing insights into dynamic crop responses. Finally, the economic feasibility and practical impact of the developed GUI-based system will be evaluated to ensure its applicability in real-world agricultural practices.

## Data Availability

The original contributions presented in the study are included in the article/supplementary material. Further inquiries can be directed to the corresponding author.
